# Genetic mutation profile of Chinese HER2-positive breast cancers and genetic predictors of responses to Neoadjuvant anti-HER2 therapy

**DOI:** 10.1007/s10549-020-05778-0

**Published:** 2020-07-07

**Authors:** Kai Li, Ning Liao, Bo Chen, Guochun Zhang, Yulei Wang, Liping Guo, Guangnan Wei, Minghan Jia, Lingzhu Wen, Chongyang Ren, Li Cao, Hsiaopei Mok, Cheukfai Li, Jiali Lin, Xiaoqing Chen, Zhou Zhang, Ting Hou, Min Li, Jing Liu, Charles M. Balch, Ning Liao

**Affiliations:** 1 Department of Breast Cancer, Guangdong Provincial People’s Hospital and Guangdong Academy of Medical Sciences, 106 Zhongshan Er Road, Guangzhou, 510080 China; 2 School of Medicine, South China University of Technology, Guangzhou, China; 3 The Second School of Clinical Medicine, Southern Medical University, Guangzhou, China; 4 Burning Rock Biotech, Guangzhou, China; 5 University of Texas MD Anderson Cancer Center, Houston, TX USA

## Abstract

**Purpose:**

Despite the therapeutic success of existing HER2-targeted therapies, tumors respond quite differently to them. This study aimed at figuring out genetic mutation profile of Chinese HER2-positive patients and investigating predictive factors of neoadjuvant anti-HER2 responses.

**Methods:**

We employed two cohorts. The first cohort was comprised of 181 HER2-positive patients treated at Guangdong Provincial People’s Hospital from 2012 to 2018. The second cohort included 40 patients from the first cohort who underwent HER2-targeted neoadjuvant chemotherapy. Genetic mutations were characterized using next-generation sequencing. We employed the most commonly used definition of pathological complete response (pCR)-eradication of tumor from both breast and lymph nodes (ypT0/is ypN0).

**Results:**

In Chinese HER2-positive breast cancer patients, TP53 (74.6%), CDK12 (64.6%) and PIK3CA (46.4%) have the highest mutation frequencies. In cohort 2, significant differences were found between pCR and non-pCR groups in terms of the initial Ki67 status, TP53 missense mutations, TP53 LOF mutations, PIK3CA mutations and ROS1 mutations (*p* = 0.028, 0.019, 0.005, 0.013, 0.049, respectively). Furthermore, TP53 LOF mutations and initial Ki67 status (OR 7.086, 95% CI 1.366–36.749, *p* = 0.020 and OR 6.007, 95% CI 1.120–32.210, *p* = 0.036, respectively) were found to be predictive of pCR status.

**Conclusion:**

TP53 LOF mutations and initial Ki67 status in HER2-positive breast cancer are predictive of pCR status after HER2-targeted NACT.

## Introduction

HER2 positivity accounts for about 15–20% of breast cancers and the development of HER2-targeted therapies has profoundly changed the course of these patients [1]. More and more HER2-targeted drugs, such as trastuzumab, pertuzumab, T-DM1 and neratinib, have become available for treatment of HER2-positive breast cancer. Despite this progress, however, many patients still die of HER2-positive breast cancer, calling for the identification and investigation of genetic profiles of HER2-positive breast cancer and predictors of responses to HER2-targeted therapies.

Neoadjuvant chemotherapy (NACT) is used commonly to downstage locally advanced cancer to allow breast-conserving surgery and to predict responses to systemic therapy based on pathological assessment. Based on the recent progress, NACT has gained momentum as an ideal setting in which to investigate predictive biomarkers of treatment responses. The Collaborative Trials in Neoadjuvant Breast Cancer (CTNeoBC) pooled analysis has confirmed NACT to be a good way to discriminate patients who have different clinical outcomes by their responses to the therapies [2]. Patients who attain complete eradication of tumor after NACT in both breast and lymph nodes have improved survival, which is defined as pathological complete response (pCR) [2, 3]. The NeoSphere and NeoALTTO trials both tried to seek higher pCR rates by different anti-HER2 combinations [4, 5] and new treatments in the past decade have significantly improved the prognosis of HER2-positive breast cancer with a pCR rate as high as 75% [6]. Despite these achievements, however, HER2-positive breast cancer patients still have a high death rate [6]. According to the latest interim analysis of the landmark KATHERINE trial, patients with HER2-positive early breast cancer who had residual invasive disease after NACT have improved survival when they receive adjuvant T-DM1 therapy after surgery [7]. As these great trials have provided effective regimens for patients with different responses, it is important to investigate the response predictors.

Large-scale, next-generation sequencing studies have provided large amounts of genetic information and produced valuable insights into the genomic landscape of primary breast cancers [8–12]. These studies have highlighted that TP53 and PIK3CA were the two most prevalent mutated genes in HER2-positive breast cancers [8] and enriched in residual tissues after HER2-targeted therapies. PIK3CA mutation rate in HER2-positive breast cancers is about 23%, and patients with PIK3CA mutations have a lower pCR rate after HER2-targeted NACT [13].

In this study, we investigated the mutation profiles of HER2-positive breast cancer patients in China and analyzed the mutation differences between primary HER2-positive breast cancers with pCR and non-pCR after HER2-targeted NACT. We hypothesized that there would be mutations predictive of the anti-HER2 therapy responses and could be used as biomarkers for guiding treatment decisions.

## Methods

### Study cohorts

This study was comprised of two cohorts. The first cohort included 181 HER2-positive patients treated at GPPH from 2012 to 2018. The second cohort included 40 patients from the first cohort who underwent HER2-targeted neoadjuvant therapy (NACT). Twenty-three patients received NACT with docetaxel 75 mg/m^2^, carboplatin (6 mg/min/ml carboplatin AUC area under curve) and trastuzumab (8 mg per kilogram intravenously as a loading dose, followed by 6 mg per kilogram intravenously every 3 weeks), while oral lapatinib was added in other 17 patients. Lapatinib was given daily at a dose of 750 mg (250 mg tablets) for the first week, followed by 1000 mg daily for a year. All 40 patients completed the scheduled 6 NACT cycles.

Detailed information of our study cohorts is listed in Table 1.

**Table 1:** Clinical characteristics of the patients in cohort 1 and cohort 2

Characteristics	Cohort 1	Cohort 2			
	Number (percentage)	Number (percentage)	PCR	Non-PCR	*p* value
Age	Median: 48, 27–79	Median: 53, 27–71	–	–	–
*Menopausal status*					
Post	78 (43.1%)	26 (65%)	12	14	0.842
Pre	103 (56.9%)	14 (35%)	6	8	
*Tumor size before NACT*					
T1-T2	170 (93.9%)	31 (77.5%)	17	14	0.052
T3-T4	11 (6.1%)	6 (22.5%)	1	8	
*TNM stage*					
I	37 (20.4%)	–	–	–	0.436
II	108 (59.7%)	24 (60%)	12	12	
III	36 (19.9%)	16 (40%)	6	10	
*HR status and HER2 status*					
HR−/HER2 +	69 (38.1%)	20 (50%)	7	13	0.204
HR + /HER2 +	112 (61.9%)	20 (50%)	11	9	
*Ki67 status*					
< 40%, +	91 (50.3%)	19 (47.5%)	12	7	**0.028**
≥ 40%, +	90 (49.7%)	21 (52.5%)	6	15	
*LN status before surgery*					
cN0	43 (23.8%)	4 (10.0%)	2	2	0.407
cN1	96 (53.0%)	22 (55.0%)	10	12	
cN2	29 (16.0%)	11 (27.5%)	6	5	
cN3	13 (7.2%)	3 (7.5%)	0	3	
*NACT regimens*					
TCH	–	18 (45.0%)	10	8	0.243
TCHL	–	22 (55.0%)	15	7	
*Tumor size after NACT*					
ypT0	–	18 (45.0%)	–	–	–
ypT1-T2	–	21 (52.5%)	–	–	–
ypT3-T4	–	1 (2.5%)	–	–	–
*LN status after surgery*					
ypN0	65 (35.9%)	26 (65%)	–	–	–
ypN1	83 (45.9%)	9 (22.5%)	–	–	–
ypN2	20 (11.0%)	2 (5%)	–	–	–
ypN3	13 (7.2%)	3 (7.5%)	–	–	–

Pathological examination of tumor specimens was performed in the Department of Pathology at GPPH. ER, PR, ROS1 and HER2 status were reconfirmed by two experienced pathologists based on IHC and fluorescence in situ hybridization (FISH) [14]. The cutoff for ER-negative and PR-negative IHC status was less than 1% staining in the nuclei. HER2 status was considered negative when an IHC score was 0 or 1 or when HER2 amplification was absent (ratio < 2.2) by FISH analysis. If any disagreements arose during the evaluation of the IHC and FISH results, a third pathologist was consulted.

### Next-generation sequencing

#### NGS library preparation

DNA fragmentation was performed using Covaris M220, followed by end repair, phosphorylation and adaptor ligation. Fragments of size 200–400 bp were selected by bead (Agencourt AMPure XP Kit, Beckman Coulter, California, USA) followed by hybridization with capture probes baits, hybrid selection with magnetic beads and PCR amplification. Subsequently, a high-sensitivity DNA assay was performed to assess the quality and size of the fragments. Indexed samples were sequenced on Nextseq500 sequencer (Illumina, Inc., California, USA) with pair-end reads.

#### Capture-based targeted DNA sequencing

Genomic profiling was performed using a panel covering 520 cancer-related genes (Burning Rock Biotech Ltd.). Among them, whole exons of 312 genes and critical exons, introns and promoter regions of the remaining 208 genes were captured.

#### Sequence data analysis

Sequence data were mapped to the human genome (hg19) using BWA aligner 0.7.10. Local alignment optimization, variant calling and annotation were performed using GATK 3.2, MuTect and VarScan. Variants were filtered using the VarScan filter pipeline, with loci with depth less than 100 filtered out. At least 5 supporting reads were needed for INDELs, while 8 supporting reads were needed for SNVs to be called. According to the ExAC, 1000 Genomes, dbSNP and ESP6500SI-V2 database, variants with population frequency over 0.1% were grouped as SNP and excluded from further analysis. Remaining variants were annotated with ANNOVAR and SnpEff v3.6. DNA translocation analysis was performed using both Tophat2 and Factera 1.4.3.

### Statistical analyses

Statistical analyses were performed using GraphPad Prism version 7.00 for Mac (GraphPad Software, La Jolla California, USA). Pearson’s Chi-square test and Yate’s continuity-corrected Chi-square test were employed for significance of differences between groups. A two-sided *p* value less than 0.05 was considered significant unless otherwise stated. To determine which covariates affected pCR, we used pathological and mutational variables by univariate and multivariate regression. We included variables which are < 0.05 for *p* value and those supposed to affect pCR status.

## Results

### Clinicopathologic features and genetic mutations of Chinese HER2-positive breast cancer patients

The patients’ clinicopathological parameters are listed in Table 1. In cohort 1, the median age was 48, and 56.9% of patients were pre-menopausal. The population was comprised of stage I, II and III patients, which account for 20.4%, 59.7% and 19.9%, respectively. The cohort 2 was derived from cohort 1, in which the median age was 53, more II, III stage patients were included and there was no difference in hormone receptor expression. No difference of pCR status was found between the two treatment groups. However, the two groups of Ki67 < 40% versus ≥ 40% had different pCR rates of 63.2% and 28.6% (*p* < 0.028) (Table 1).

**Fig. 1:**
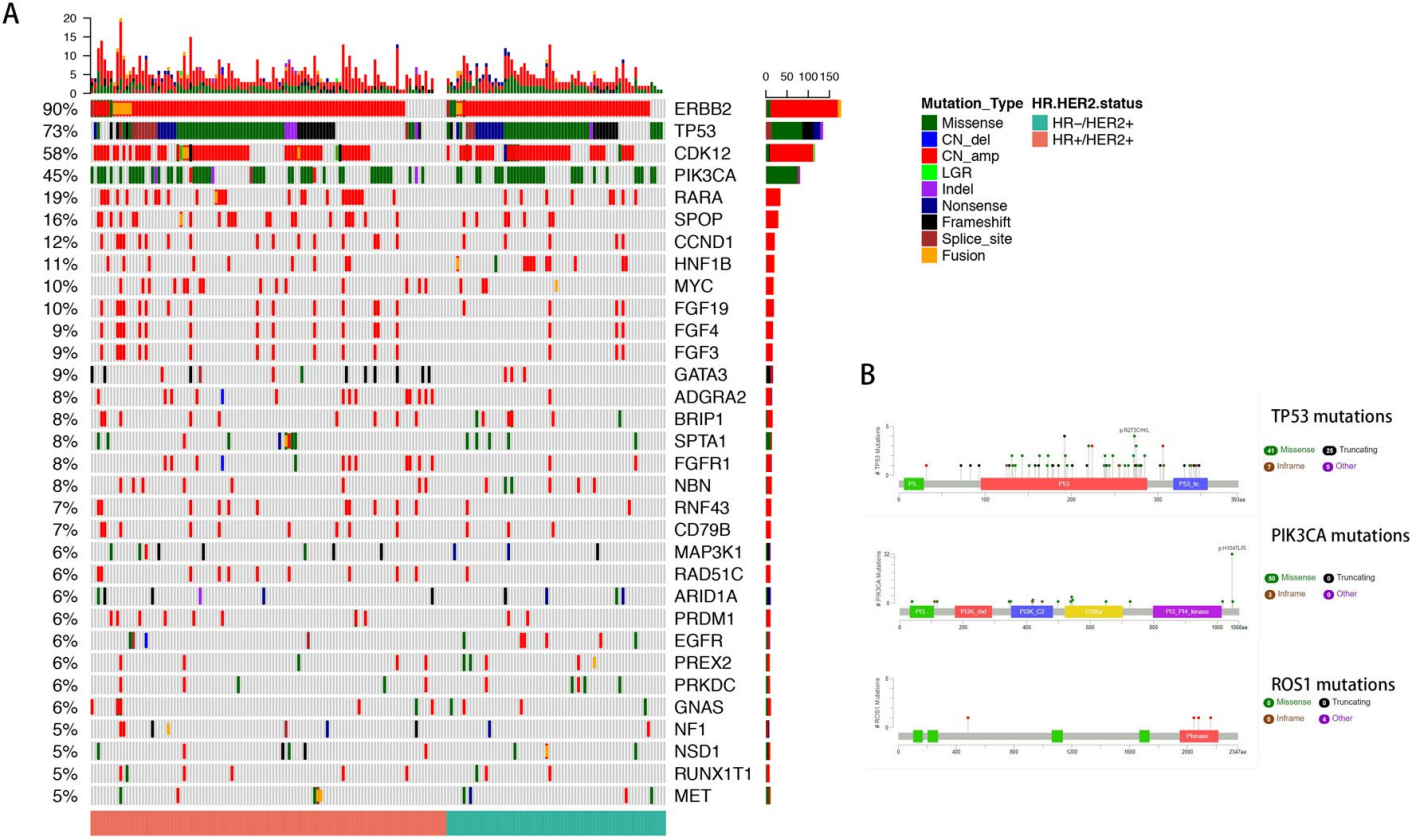
The mutational landscape of 181 Chinese patients with HER2-positive breast cancer (cohort 1) elucidated using a 520-gene panel (OncoScreen Plus, Burning Rock Biotech). **a** Oncoprint summarizing the mutational landscape of the cohort. Only somatic alterations with a frequency of 5% or greater in the whole cohort are displayed. The HR status of the patients was indicated at the bottom of the oncoprint, wherein green represents patients with HR-negative (HR−/HER2+) status and red represents patients with HR-positive (HR+/HER2+) status. Each column represents a patient and each row represents a gene. Numbers on the left represent the percentage of patients with mutations in a specific gene. Top plot represents the overall number of mutations a patient carried. Different colors denote different types of mutations. **b** Lollipop diagrams depicting the type and specific locations of TP53, PIK3CA and ROS1 mutations in cohort 2. Colored boxes depict the different functional domains along the gene. Colored circles denote the type of mutation, while the location of the circle specifies the mutation site. The length of the lollipop represents the number of patients harboring a specific variant. The legend on the right side summarizes the total number of mutation types such as missense, truncating, inframe and other mutations

**Fig. 2:**
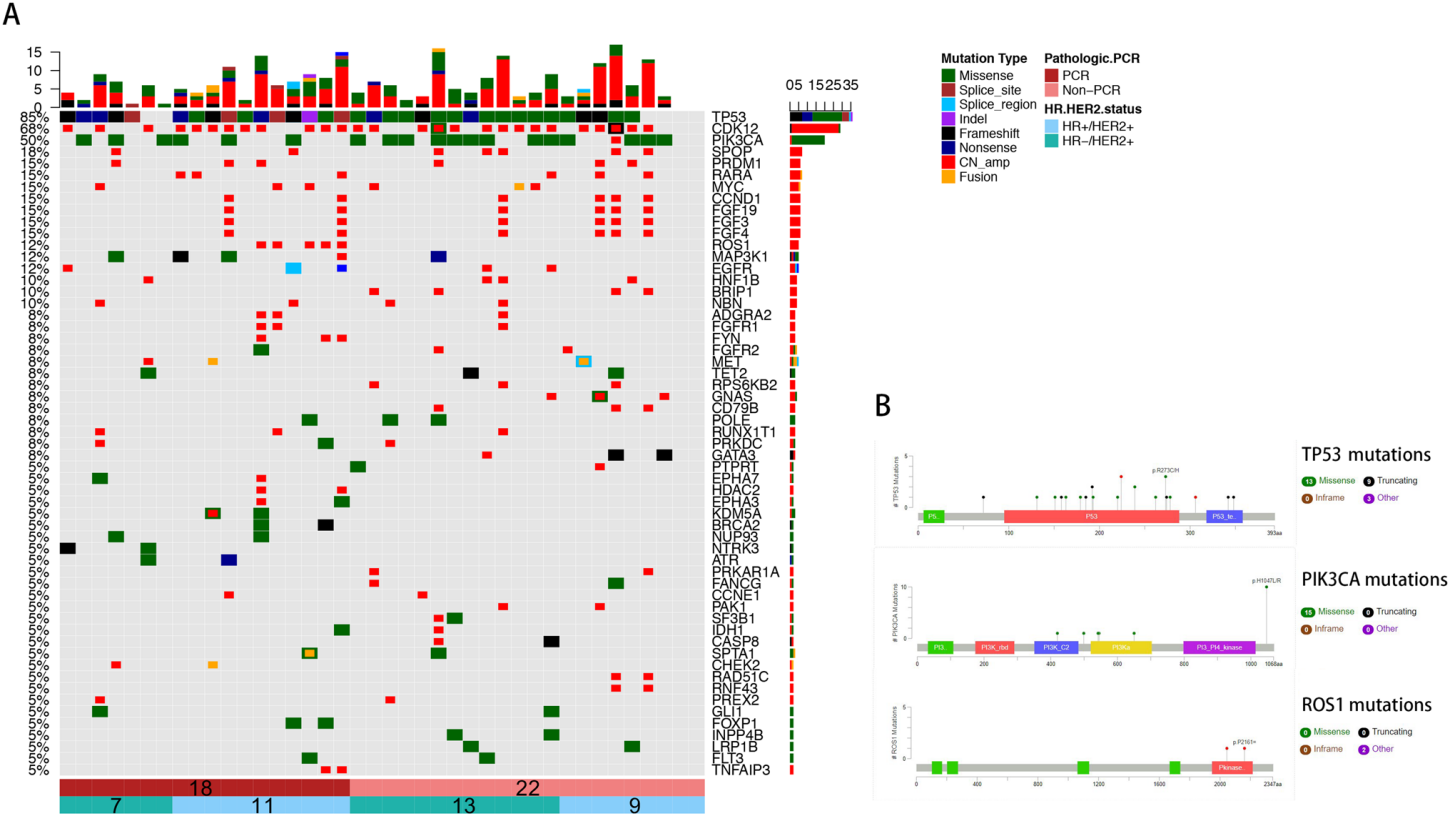
The mutational landscape of 40 Chinese patients with early-stage HER2-positive breast cancer who received HER2 inhibitors as neoadjuvant therapy (cohort 2) elucidated using a 520-gene panel (OncoScreen Plus, Burning Rock Biotech). **a** Oncoprint summarizing the mutational landscape of the cohort. Only somatic alterations with a frequency of 5% or greater in the whole cohort are displayed. The pathologic complete response (pCR) and HR status of the patients were indicated at the bottom of the oncoprint, wherein red represents patients achieving pCR (*n* = 18), pink represents patients with non-pCR (*n* = 22); cyan represents patients with HR-positive (HR+/HER2+) status (pCR, *n* = 11; non-pCR, *n* = 9) and green represents patients with HR-negative (HR−/HER2+) status (pCR, *n* = 7; non-pCR, *n* = 13). Each column represents a patient and each row represents a gene. Numbers on the left represent the percentage of patients with mutations in a specific gene. Top plot represents the overall number of mutations a patient carried. Different colors denote different types of mutations. **b** Lollipop diagrams depicting the type and specific locations of TP53, PIK3CA and ROS1 mutations in cohort 2. Colored boxes depict the different functional domains along the gene. Colored circles denote the type of mutation, while the location of the circle specifies the mutation site. The length of the lollipop represents the number of patients harboring a specific variant. The legend on the right side summarizes the total number of mutation types such as missense, truncating, inframe and other mutations

We next analyzed the mutation profiles in Chinese HER2-positive breast cancers (Fig. 1 and Table 2). The most commonly mutated genes in Chinese HER2-positive breast cancer patients were TP53 (74.6%), CDK12 (64.6%) and PIK3CA (46.4%). Most of the TP53 mutations were missense mutations (40.35%) and LOF mutations (21.5%). Interestingly, the ROS1 mutations were only detected in HR + HER2 + patients. Mutation sites of TP53, PIK3CA and ROS1 are listed in Fig. 1. No TP53 mutation hotspots were found in these Chinese HER2 + patients, while p.H1047R was investigated to be hotspot in PIK3CA mutations (Fig. 1 and Table 3). In cohort 2 (Table 2 and Fig. 2), TP53 (90.0%), CDK12 (77.5%) and PIK3CA (55%) were still the most mutated genes. And 5 patients got ROS1 mutations, all of whom belong to HR + HER2 + subgroup. The mutation sites of TP53, PIK3CA and ROS1 in cohort 2 are also listed in Fig. 2.

**Table 2:** Associations of somatic alterations with pCR or non-pCR

Mutations		Cohort 1			Cohort 2				
		HR+/HER2+ (*n*/181)	HR-/HER2 + (*n*/181)	Total (*n*/181)	HR+/HER2 + (n/40)	HR-/HER2 + (*n*/40)	pCR (*n*/18)	Non-pCR (*n*/22)	*p* value
TP53	Missense	40 (22.1%)	33 (18.2%)	73 (40.35%)	7 (17.5%)	10 (25.0%)	4 (22.2%)	13 (59.1%)	**0.019**
	LOF mutations	27 (14.9%)	12 (6.6%)	39 (21.5%)	7 (17.5%)	10 (25.0%)	12 (66.7%)	5 (22.7%)	**0.005**
	Splice_acceptor	7 (3.9%)	1 (0.6%)	8 (4.5%)	0	2 (5.0%)	2 (11.1%)	0	**–**
	Splice_donor	3 (1.7%)	2 (1.1%)	5 (2.8%)	1 (2.5%)	1 (2.5%)	2 (11.1%)	0	**–**
	Frame shift	17 (9.4%)	9 (5.0%)	26 (14.4%)	4 (10.0%)	3 (7.5%)	4 (22.2%)	3 (13.6%)	0.770*
	Nonsense(stop gain)	7 (3.9%)	10 (5.5%)	17 (9.4%)	2 (10.0%)	4 (10.0%)	4 (22.2%)	2 (9.1%)	0.476*
	Splice_region	0	1 (0.6%)	1 (0.6%)	1 (2.5%)	0	1 (5.5%)	0	**–**
	Indel	5 (2.8%)	1 (0.6%)	6 (7.4%)	1 (2.5%)	0	1 (5.5%)	0	**–**
	Total	79 (43.6%)	56 (30.9%)	135 (74.6%)	16 (40.0%)	20 (50.0%)	18 (88.9%)^#^	18 (81.8%)	0.859*
CDK12	CN_amp	60 (33.1%)	41 (22.7%)	101 (55.8%)	14 (35.0%)	13 (37.5%)	13 (72.2%)	14 (63.6%)	1.000*
	Missense	1 (0.6%)	6 (3.3%)	7 (3.9%)	0	1 (2.5%)	0	1 (4.5%)	–
	Frameshift_variant	2 (1.1%)	0	2 (1.1%)	1 (2.5%)	0	0	1 (4.5%)	–
	Fusion	4 (2.2%)	0	4 (2.2%)	–	–	–	–	–
	LGR	2 (1.1%)	0	2 (1.1%)	–	–	–	–	–
	Total	69 (38.1%)	48 (26.5%)	117 (64.6%)	15 (37.5%)	14 (40%)	13 (72.2%)	16 (72.7%)	1.000*
PIK3CA	Missense	49 (27.1%)	30 (16.6%)	79 (43.6%)	9 (22.5%)	12 (30.0%)	6 (33.3%)	15 (68.2%)	–
	CN_amp	2 (1.1%)	0	2 (1.1%)	1 (2.5%)	0	0	1 (4.5%)	–
	Indel	3 (1.7%)	0	3 (1.7%)	–	–	–	–	–
	Total	54 (29.8%)	30 (16.6%)	84 (46.4%)	10 (25%)	12 (30.0%)	6 (33.3%)	16 (72.7%)	**0.013**
RARA	CN_amp	26 (14.4%)	8 (4.4%)	34 (18.8%)	6 (15.0%)	2 (5.0%)	5 (27.8%)	3 (13.6%)	0.475*
	Fusion	1 (0.6%)	0	1 (0.6%)	–	–	–	–	–
	Total	27 (14.9%)	8 (4.4%)	35 (19.3%)	6 (15.0%)	2 (5.0%)	5 (27.8%)	3 (13.6%)	0.475*
SPOP	CN_amp	22 (12.2%)	7 (3.9%)	29 (16.0%)	3 (15.0%)	4 (10.0%)	2 (11.1%)	5 (22.7%)	0.587*
	Fusion	1 (0.6%)	0	1 (0.6%)	–	–	–	–	–
	Total	23 (12.7%)	7 (3.9%)	30 (16.6%)	3 (15.0%)	4 (10.0%)	2 (11.1%)	5 (22.7%)	0.587*
CCND1	CN_amp	16 (8.9%)	5 (2.8%)	21 (11.6%)	5 (12.5%)	1 (2.5%)	2 (11.1%)	4 (18.2%)	0.859*
Myc	CN_amp	15 (8.3%)	3 (1.7%)	18 (9.9%)	2 (10.0%)	3 (7.5%)	3 (16.6%)	2 (9.1%)	1.000*
	Fusion	0	1 (0.6%)	1 (0.6%)	0	1 (2.5%)	0	1 (4.5%)	–
	Total	15 (8.3%)	4 (2.2%)	19 (10.5%)	2 (10.0%)	4 (10.0%)	3 (16.6%)	3 (13.6%)	1.000*
FGF19	CN_amp	15 (8.3%)	4 (2.2%)	19 (10.5%)	5 (12.5%)	1 (2.5%)	2 (11.1%)	4 (18.2%)	0.859*
FGF3	CN_amp	13 (7.2%)	3 (1.7%)	16 (8.8%)	5 (12.5%)	1 (2.5%)	2 (11.1%)	4 (18.2%)	0.859*
FGF4	CN_amp	14 (7.7%)	3 (1.7%)	17 (9.4%)	5 (12.5%)	1 (2.5%)	2 (11.1%)	4 (18.2%)	0.859*
ROS1	CN_amp	6 (3.3%)	0	6 (3.3%)	5 (12.5%)	0	5 (27.8%)	0	0.049*
	Splice_site	1 (0.6%)	0	1 (0.6%)	0	0	0	0	**–**
	Total	7 (3.9%)	0	7 (3.9%)	5 (12.5%)	0	5 (27.8%)	0	0.049*

Bold indicates the significance of *p* value < 0.05

^#^18 mutations belong to 16 of the patients with pCR

*Indicates analyzed by Continuity Correction of Pearson’s Chi-square test, while other *p* values were got by Pearson's Chi-square test

**Table 3:** Mutations of TP53 and PIK3CA in cohort 2

Gene	Mutation_type	Exon_rank	Description	AF (%)	CHROM	POS	REF	ALT	Patient.count
TP53	missense_variant	5	p.A159V	12.57	17	7,578,454	G	A	1
	missense_variant	8	p.G262V	38.51	17	7,577,153	C	A	1
	missense_variant	5	p.H179R	29.08	17	7,578,394	T	C	1
	missense_variant	6	p.H193L	27.33	17	7,578,271	T	A	1
	missense_variant	5	p.N131I	9.18	17	7,578,538	T	A	1
	missense_variant	7	p.N239D	47.06	17	7,577,566	T	C	1
	missense_variant	5	p.P151S	56.70	17	7,578,479	G	A	1
	missense_variant	8	p.P278R	29.25	17	7,577,105	G	C	1
	missense_variant	8	p.P278S	11.58	17	7,577,106	G	A	1
	missense_variant	6	p.R209S	37.70	17	7,578,222	T	G	1
	missense_variant	8	p.R273C	3.18	17	7,577,121	G	A	1
	missense_variant	8	p.R273H	10.65	17	7,577,120	C	T	2
	missense_variant	8	p.R282W	19.20	17	7,577,094	G	A	1
	missense_variant	5	p.Y126D	21.26	17	7,578,554	A	C	1
	missense_variant	5	p.Y163C	39.06	17	7,578,442	T	C	1
	missense_variant	6	p.Y220N	46.71	17	7,578,191	A	T	1
	conservative_inframe_deletion	6	p.F212_S215del	31.38	17	7,578,203	CACTATGTCGAAAA	CT	1
	frameshift_variant	10	p.L348fs	6.11	17	7,573,966	TGGGCATCCTTGAGTTCCAAG	T	1
	frameshift_variant	4	p.L93fs	37.73	17	7,579,408	CA	C	1
	frameshift_variant	7	p.N239fs	30.55	17	7,577,564	GT	G	1
	frameshift_variant	4	p.P72fs	17.93	17	7,579,470	CGGG	CGC	1
	frameshift_variant	5	p.R158fs	28.17	17	7,578,445	ATGGCCATGGCGCG	A	1
	frameshift_variant	5	p.S185fs	22.44	17	7,578,373	TCGC	TT	1
	frameshift_variant	8	p.V274fs	25.05	17	7,577,118	C	CA	1
	splice_acceptor_variant	9	c.920-1G>A	39.53	17	7,576,927	C	T	1
	splice_acceptor_variant	9	p.S261_G262delins???	16.55	17	7,577,151	TACCACTACTCAGGATAGGAAAAG	TT	1
	splice_donor_variant	6	c.672 + 1G>A	11.68	17	7,578,176	C	T	1
	splice_donor_variant	6	c.672 + 1G>T	9.46	17	7,578,176	C	A	1
	splice_region_variant	6	p.E224D	26.46	17	7,578,177	C	A	1
	stop_gained	5	p.Q144*	8.93	17	7,578,500	G	A	1
	stop_gained	6	p.Q192*	58.54	17	7,578,275	G	A	3
	stop_gained	10	p.R342*	35.58	17	7,574,003	G	A	1
	stop_gained	7	p.Y236*	36.95	17	7,577,573	G	T	1
PIK3CA	missense_variant	21	p.H1047R	6.73	3	178,952,085	A	G	13
	missense_variant	8	p.C420R	15.24	3	178,927,980	T	C	1
	missense_variant	10	p.E542K	17.26	3	178,936,082	G	A	1
	missense_variant	10	p.E545G	14.01	3	178,936,092	A	G	1
	missense_variant	21	p.H1047L	37.45	3	178,952,085	A	T	1
	missense_variant	5	p.N345K	10.64	3	178,921,553	T	A	2
	missense_variant	9	p.S499F	4.89	3	178,928,310	C	T	1
	missense_variant	13	p.V650M	11.69	3	178,937,773	G	A	1
	cn_amp	NA	cn_amp	3.87	3q26.32	3q26.32	19	17	1

### Genetic mutation difference between HER2-positive breast cancer patients with and without pCR

In the NACT group cohort 2, mutations were frequently identified in 48 genes shown in the oncoprint (Fig. 2) and some are selected in Table 2. The genetic mutation profiles were quite different between HER2-positive breast cancer patients with and without pCR. In pCR group, mutation rates of the most frequently mutated genes, such as TP53, PIK3CA, CDK12, SPOP, FGF3, FGF4 and FGF19, were 88.9%, 33.3%, 72.2%, 11.1%, 11.1%, 11.1% and 11.1%, while in non-pCR group their mutation rates were 81.8%, 72.7%, 72.7%, 22.7%, 18.2%, 18.2% and 18.2%.

Then we detected the differences between the pCR and non-pCR groups according to pathological and mutational variables and found significant differences in terms of the initial Ki67 status, TP53 missense mutations, TP53 LOF mutations, PIK3CA mutations and ROS1 mutations (*p* = 0.028, 0.019, 0.005, 0.013, 0.049, respectively, Tables 1, 2).

One PIK3CA mutation hotspot was examined to be p.H1047R which belongs to be a missense mutation. Three mutation sites (p.V650M, p.E545G and p.E542K) were detected to be located in the PI3Ka subunit which has been found to be crucial for PIK3CA function.

**Fig. 3:**
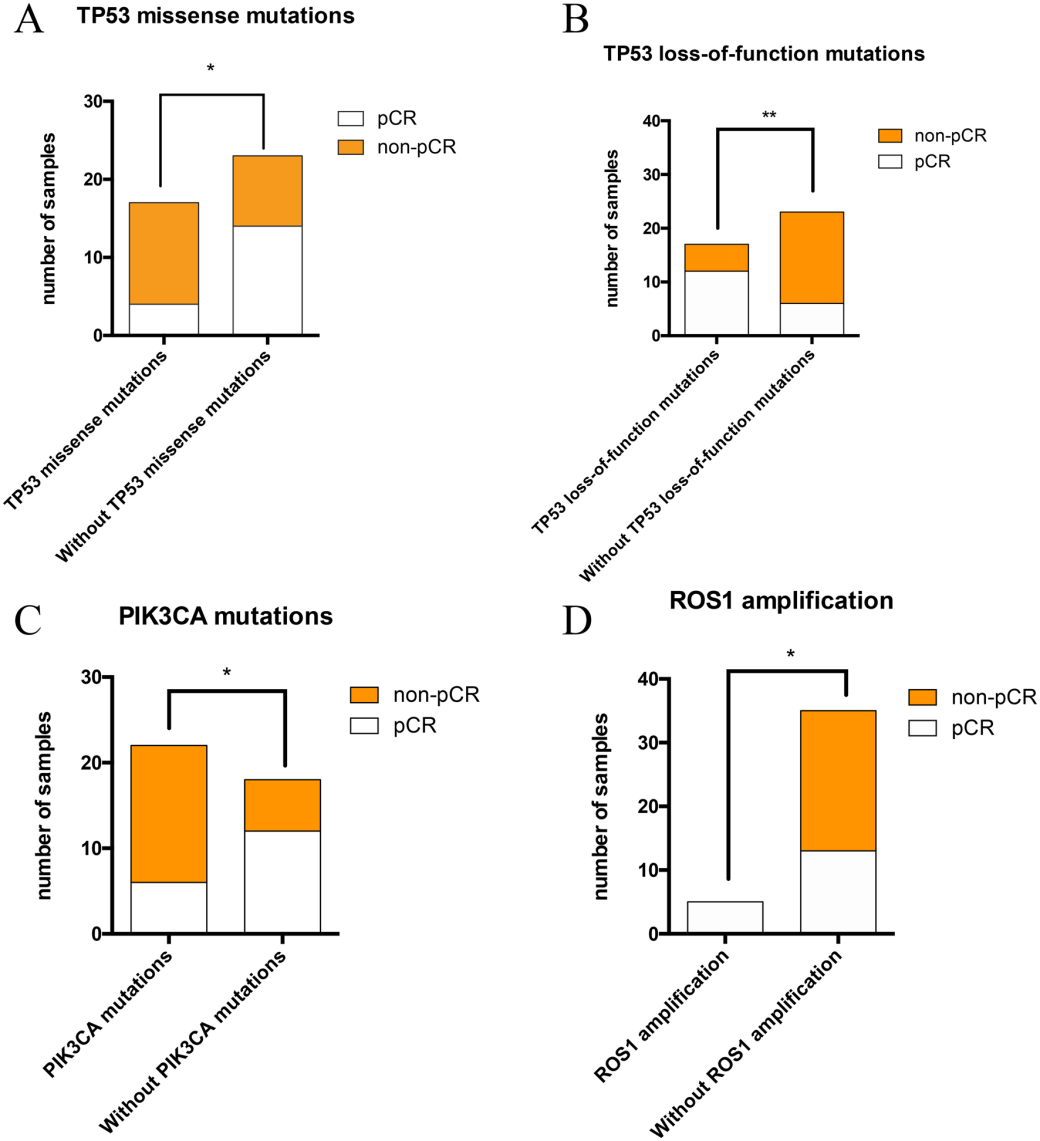
Differences between pCR and non-pCR groups in terms of genetic mutations. **a**–**d** Pearson’s Chi-square test and Yate’s continuity-corrected Chi-square test analyzed the associations of TP53 missense and LOF mutations, PIK3CA mutations and ROS1 amplifications with pCR or non-pCR of HER2-positive breast cancers after NACT. *Indicates 0.01 < *p* < 0.05, **indicates *p* < 0.01

There was a significant difference of PIK3CA mutation frequency between pCR and non-pCR group (33.3% vs 72.7%; *p* = 0.013, Table 2 and Fig. 3).

**Fig. 4:**
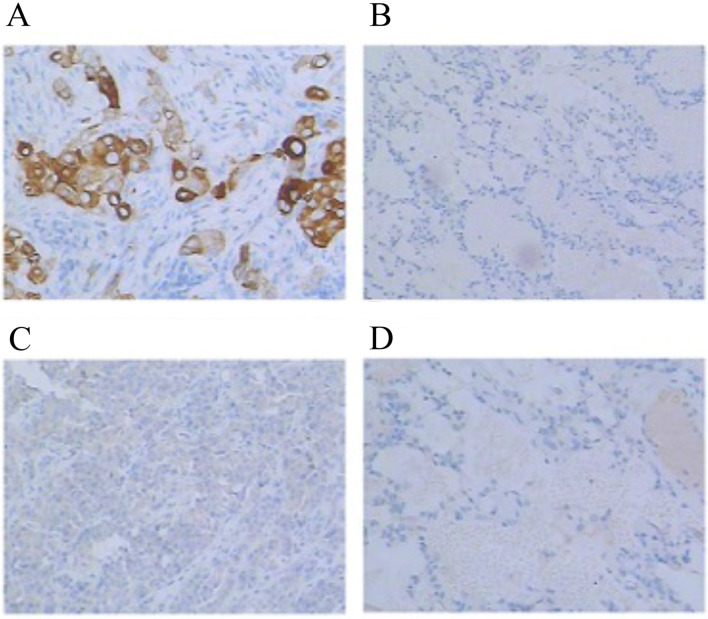
ROS1 expression in protein level. **a**, **b** Positive and negative control of ROS1 expression by IHC. **c** ROS1 expression in samples with ROS1 amplification (negative). **d** ROS1 expression in samples without ROS1 amplification (negative)

ROS1 amplification was only investigated in 5 hormone receptor-positive patients who all got pCR. The ROS1 amplification breast cancers were found to have higher pCR rate (*p* = 0.049) (Table 2 and Fig. 2). However, when we go further to investigate whether ROS1 had amplification in protein level by immunohistochemistry, the result was negative (Figs. 3, 4).

### Predictors for pCR or non-pCR

As shown above, some mutations and pathological factors seemed to affect pCR status and, to determine which of those are the predictive factors, we did univariate and multivariate regression. For pCR status, three covariates were significant in the univariate analyses (Tumors size, TP53 missense mutations, TP53 LOF mutations), but only one was retained and a new one appeared after forward selection in the multivariate analysis—TP53 LOF mutations and initial Ki67 status (OR 7.086, 95% CI 1.366–36.749, *p* = 0.020 and OR 6.007, 95% CI 1.120–32.210, *p* = 0.036, respectively, in Table 4). Some factors selected in the univariate model seemed to be highly correlated and for this reason were rejected or appeared in the multivariate model.

**Table 4:** Predictors of pCR status analyzed by univariate and multivariate regression

Factors	Univariate regression		Multivariate regression	
	OR (95% CI)	*p* value	OR (95% CI)	*p* value
Tumor size	9.714 (1.081–87.313)	**0.042**	11.844 (0.993–141.214)	
Hormone receptor status	0.636 (0.180–2.251)	0.483		
Initial Ki67 status	3.500 (0.945–12.966)	0.061	6.007 (1.120–32.210)	**0.036**
NACT regimen	0.467 (0.129–1.692)	0.246		
TP53 missense mutations	0.198 (0.049–0.801)	**0.023**		
TP53 LOF mutations	6.800 (1.680–27.522)	**0.007**	7.086 (1.366–36.749)	**0.020**
PIK3CA mutations	0.286 (0.077–1.058)	0.061		
ROS1 mutations	8.077 (0.846–77.070)	0.07		

Bold indicates the significance of *p* value < 0.05

TP53 missense mutations, TP53 LOF mutations and tumor size were significantly associated with pCR status by univariate regression; however, only TP53 LOF mutations retained when multivariate regression was carried out, and Initial Ki67 status turned out to be predictive

## Discussion

Our findings indicate significant associations (*p* = 0.020 and *p* = 0.036) of TP53 LOF mutations and lower initial Ki67 status (< 40%) with a high probability of pCR in HER2-positive breast cancer patients receiving NACT.

TP53 has been reported to have heterogenous types of mutations which include attenuation of function, separation of function or neomorphic function [15–17]. There are gain-of-function mutations mostly TP53 missense mutations and LOF mutations which have a common characteristic of losing functions of wild-type TP53 and composed of splice site, frame shift and nonsense mutations [16, 18].

HER2-positive breast cancer has a high frequency of TP53 mutations (up to 72%) [8], and in our previous study, the mutation frequency of TP53 in the triple-positive HR + /HER2 + group is 66.1% and up to 89.3% in the HR-/HER2 + group in Chinese breast cancer patients [19]. Interestingly, the TP53 mutation rate was similar in those of HER2-positive patients who achieved a pCR or not (88.9% v 81.8%), and there was no statistical difference of TP53 mutation rate between two groups (*p* = 0.859). However, as far as specific types of TP53 mutations were considered, such as missense mutations, LOF mutations and so on, quite significant mutational differences were manifested. TP53 missense mutation rates in pCR and non-pCR groups were quite different (22.2% vs 59.1%, *p* = 0.019, Table 2 and Fig. 3). Similarly, patients in pCR group were detected to have much higher LOF mutation rate (66.7% vs 22.7%, *p* = 0.005, Table 2 and Fig. 3). Univariate logistic regression showed TP53 missense mutation and TP53 LOF mutation were significantly associated with pCR status (OR 0.198, 95% CI 0.049–0.801, *p* = 0.023 and OR 6.800, 95% CI 1.680–27.522, *p* = 0.007). However, when multivariate logistic regression was applied, only TP53 LOF mutation was retained to be predictive of pCR status.

And why is that? How TP53 LOF mutation mediates a good response to NACT is complicated. TP53 has been reported to be activated in response to mitotic stress caused by agents such as taxanes that disrupt microtubules [20]. Evidences from breast cancer models and tumors have shown p53 directs cells to undergo cell cycle arrest and senescence [21, 22]. One mechanism that contributes to senescent cell survival and persistence in the residual disease after chemotherapy treatment is the engulfing and cannibalizing of neighboring cells [23]. We believe that TP53 LOF mutations lose capacity to activate cell cycle arrest and senescence to escape apoptosis induced by therapy, and as a result mediate a good response to NACT.

Some TP53 missense mutations are associated with enhanced characteristics of invasion and metastasis when they acquire a gain-of-function effect [24]. More than 80% of TP53 alterations are missense mutations that will produce a stable but transcriptionally deficient protein. These mutant-TP53-expressing tumors are aggressive and associated to poor prognosis [25, 26]. Compelling evidences have proved that TP53 missense mutations promote cell migration and metastasis and dramatically influence tumor progression [27–29]. In our study, TP53 missense mutations showed significant difference between pCR and non-pCR groups, and also a predictor for non-pCR by univariate regression. We speculated that TP53 missense may affect therapy response depending on other variables.

TP53 mutations were not predictive of neoadjuvant chemotherapy response in the EORTC 10,994/BIG 1-00 trial [30]. However, in that study, patients with HER2 + breast cancers were randomly assigned to different NACT groups without HER2-targeted therapy, and the yeast assay was used to assess TP53 mutations which does not distinguish between pure loss-of-function mutations compared to mutations with simultaneous gain and loss-of-function [31, 32]. Several molecular alterations are thought to contribute to trastuzumab resistance, including TP53 mutation [33, 34] and PIK3CA alteration [13, 35, 36], but results evaluating these biomarkers as response predictors have been inconsistent.

Two retrospective studies [37, 38] reported TP53 mutations were significantly predictive of HER2 + patient treatment response (pCR) to neoadjuvant chemotherapies. Soley et al. report that for patient samples with concordant BluePrint/MammaPrint and PAM50 data, the pCR plus non-pCR rate among patients whose tumors were TP53 mutant was 17/39 (44%), whereas in patients whose tumors were TP53 wild type, it was 5/31 (16%), *p* = 0.020 [37]. And Stefan et al. report that the response rate among TP53-mutated patients was 30%, significantly higher than TP53 wild-type patients (10%; *p* = 0.0032) [38]. However, both studies used the AmpliChip TP53 assay (Roche Molecular Systems, Pleasanton, CA), a DNA microarray-based resequencing assay designed to detect single-base substitutions and single-base deletions in all coding regions of the TP53 gene, which needs a reference sequence and is unable to detect all the possible mutations like a NGS assay [39, 40]. Two other studies found no associations of TP53 mutations with HER2 + NACT treatment response [41, 42]. However, all these studies only examined associations of TP53 mutations as a whole with treatment response, despite the fact that TP53 missense mutations and loss-of-function mutations have quite different functions during breast cancer progression [16, 24, 26]. In our study, we assessed the associations of TP53 missense mutations and LOF mutations separately with treatment response and found quite different predictive characteristics.

From a tumor biological point of view, Ki67 should be viewed as a continuous variable, as it reflects the percentage of proliferating cells in the tumor, which can reach any value between 0 and 100%. The fact of defining our cut points should not be seen as a limitation of the marker but point to a strength of Ki67, as studies have shown a wide range of cut points was significant for various endpoints and subgroups [43]. Therefore in our study, Ki67 is still a predictive biomarker for HER2-positive breast cancer subgroup receiving chemotherapy and HER2-targeted therapy.

Tumor size was analyzed to be a predictor of pCR status by univariate regression and not by multivariate regression. It is easy to understand that tumor size is a factor susceptible to other pathological and mutational factors.

In conclusion, our study reports TP53 LOF mutations and initial Ki67 status predict pCR status for HER2-positive breast cancer patients receiving NACT. As this study is an exploratory retrospective study of small size, further prospective clinical research with large sample is still needed.
